# Triple-balloon occlusion for targeted retrograde coronary venous ethanol ablation of left ventricular summit ventricular tachycardia

**DOI:** 10.1016/j.hrcr.2026.03.015

**Published:** 2026-03-24

**Authors:** Mehmet Özgeyik, Mehmet Semih Belpinar, Ramazan Anıl Eşki, Ibrahim Etem Dural, Basar Candemir

**Affiliations:** 1 TCSB Eskişehir City Hospital, Eskişehir, Turkey; 2 Faculty of Medicine, Afyonkarahisar Health Sciences University, Afyonkarahisar, Turkey; 3 Faculty of Medicine, Ankara University, Ankara, Turkey

**Keywords:** Left ventricular summit, Ventricular arrhythmia, Retrograde coronary venous ethanol ablation, Triple-balloon occlusion technique, Intramural substrate, Bailout ablation strategy

**Key Teaching Points**

• Ventricular arrhythmias originating from the left ventricular summit are frequently resistant to conventional endocardial radiofrequency ablation because of their epicardial location and complex intramural substrate.
• Retrograde coronary venous ethanol ablation represents an effective bailout strategy when previous endocardial and epicardial approaches fail.
• Intervenous collaterals may permit unintended ethanol spillover; multiballoon occlusion techniques can enhance ethanol confinement and improve lesion specificity.
• A triple-balloon technique may allow precise isolation of a predefined venous segment in complex coronary venous anatomy, facilitating targeted substrate modification while minimizing collateral myocardial injury.

## Introduction

Radiofrequency (RF) catheter ablation is a guideline-recommended first-line therapy for drug-refractory ventricular arrhythmias (VAs).^1^^,^^2^ Despite its efficacy, procedural failure may occur owing to inadequate catheter–myocardial contact, anatomic barriers such as coronary arteries, prosthetic valves or phrenic nerve proximity, and deep intramural substrates inaccessible to conventional unipolar RF energy.^3^, ^4^, ^5^ To overcome these limitations, alternative strategies including bipolar RF ablation, needle ablation, stereotactic radioablation, half-normal saline irrigation, and ethanol ablation have been developed.^6^, ^7^, ^8^, ^9^, ^10^

Ethanol infusion induces myocardial necrosis through rapid cellular membrane disruption and protein denaturation. Both transarterial and transvenous approaches have been described for refractory ventricular substrates.^11^ Retrograde coronary venous ethanol ablation was developed to mitigate limitations of transarterial ethanol delivery such as coronary artery dissection, uncontrolled spillover, and rapid arteriovenous washout.

The left ventricular summit (LVS) represents the most superior epicardial portion of the basal left ventricular free wall, anatomically bounded by the left anterior descending and circumflex arteries. VAs originating from this region are frequently resistant to endocardial RF ablation owing to epicardial location and intramural depth.

However, intervenous collaterals may limit effective ethanol delivery by permitting distal leakage, thereby reducing lesion specificity and increasing the risk of nontarget myocardial injury. Double-balloon occlusion techniques have been described to improve localization.^12^ Here, we present a novel triple-balloon technique to enhance ethanol confinement in a patient with RF-refractory LVS ventricular tachycardia (VT).

## Case report

**Figure 1:**
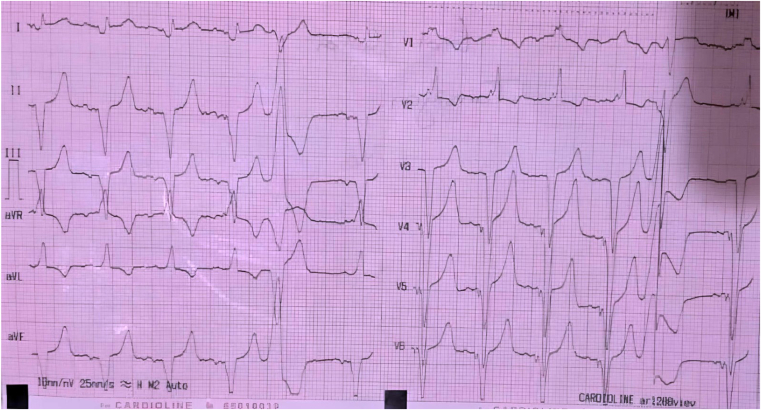
Electrocardiogram of a ventricular premature beat consistent with left ventricular summit area.

A 66-year-old man with previous surgical aortic valve replacement and nonischemic cardiomyopathy presented with drug-refractory VT and recurrent implantable cardioverter-defibrillator shocks. Surface electrocardiography demonstrated ventricular ectopy consistent with LVS origin (Figure 1). Endocardial ablation via transseptal approach had previously been attempted. Despite high-power, long-duration lesions delivered both endocardially and within distal coronary venous branches, VT recurred. Epicardial access was unsuccessful owing to dense pericardial adhesions.

The patient was scheduled for retrograde coronary venous ethanol ablation during a subsequent procedure. Using the CARTO 3-dimensional mapping system (Biosense Webster, Diamond Bar, CA) with a high-density Pentaray catheter introduced via transseptal access, the earliest endocardial activation was recorded at the anteromedial mitral annular region, adjacent to the presumed epicardial LVS origin. Diastolic activity was limited, suggesting an intramural or epicardial substrate.

**Figure 2:**
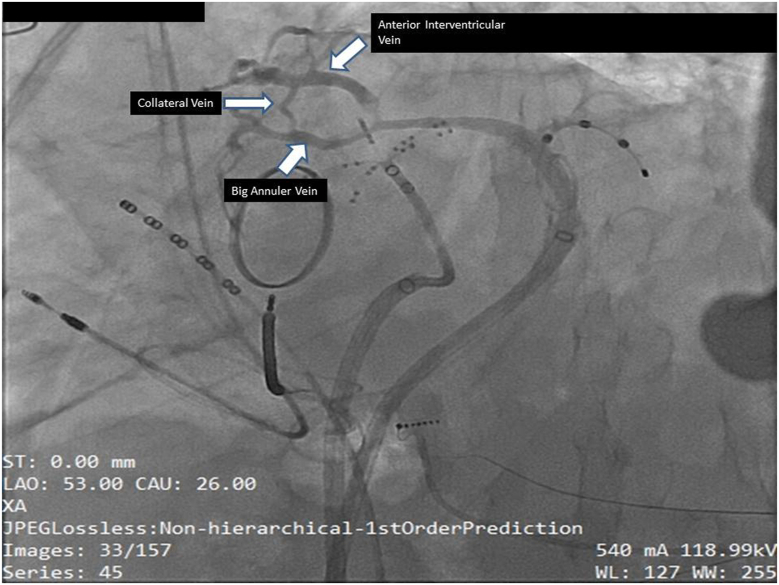
Venous anatomy of distal coronary sinus (LAO 53° and caudal 26° projection). LAO = left anterior oblique.

**Figure 3:**
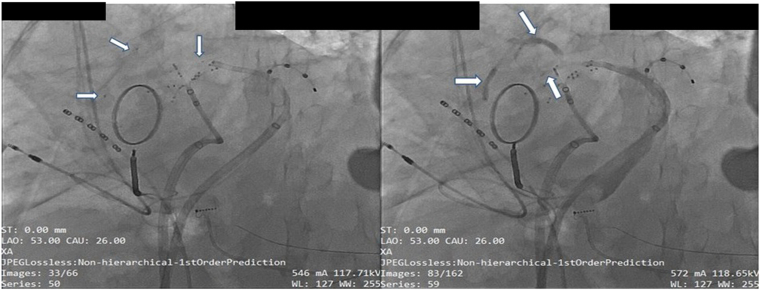
*White arrows* show the balloons’ positions (LAO 53° and caudal 26° projection) The catheter visualized in the fluoroscopic images represents the previously implanted CRT left ventricular pacing lead within the coronary venous system and not a mapping microcatheter. CRT = cardiac resynchronization therapy; LAO = left anterior oblique.

The coronary venous system was cannulated using a bidirectional steerable sheath (Agilis, Abbott, Plymouth, MN). A JR4 guiding catheter was advanced to the junction of the great cardiac vein and anterior interventricular vein (AIV). Venography revealed a large annular vein supplying the basal septal region with an intercommunicating collateral draining into the AIV (Figure 2). To restrict ethanol delivery to the region opposite the presumed VT exit site, 2 guidewires were advanced into the distal annular vein and 1 into the collateral branch. A semicompliant over-the-wire balloon (2.5 × 15 mm) was positioned proximally at the great cardiac vein–AIV junction. A monorail balloon was positioned distally in the annular vein, and a second monorail balloon (1.5 × 12 mm) was placed within the collateral branch (Figure 3).

Dedicated venous activation mapping or pace mapping using a slim multipolar catheter or unipolar wire mapping was not performed owing to a lack of resources.

**Figure 4:**
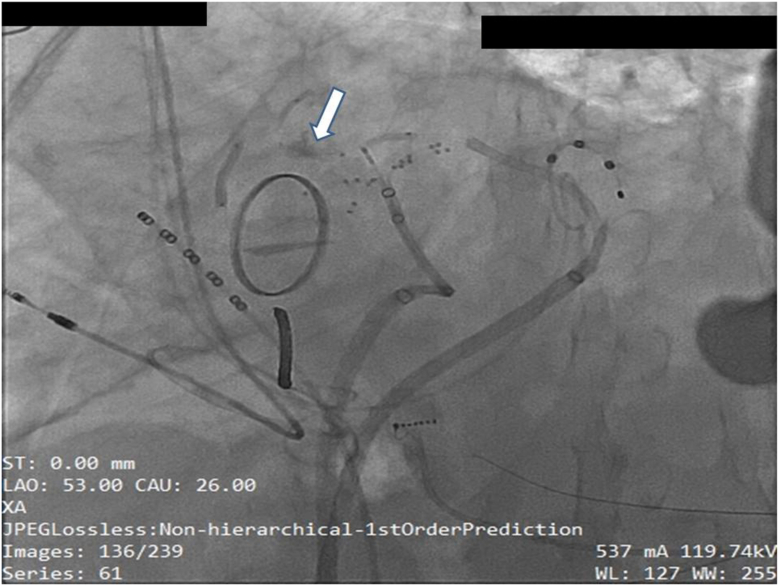
Myocardial staining after ethanol infusion (LAO 53° and caudal 26° projection). LAO = left anterior oblique.

**Figure 5:**
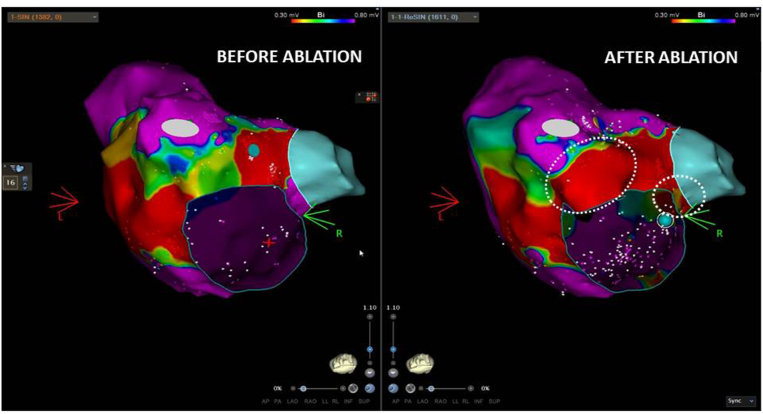
Preablation and postablation voltage maps with projected mapping points displayed to illustrate local point density and spatial sampling distribution. Total point counts reflect all points acquired for the entire chamber geometry.

After confirming complete occlusion with contrast injection, ethanol was slowly administered through the proximal balloon (3 injections of 2 mL each). Progressive myocardial staining was observed after each injection (Figure 4). Postethanol electroanatomic mapping demonstrated increased bipolar scar in the targeted region (Figure 5), and VT was no longer inducible.

Peak high-sensitivity cardiac troponin I reached 21 ng/L (institutional upper reference limit 14 ng/L) on day 2 and subsequently declined. No clinical or electrocardiographic evidence of ischemia was observed. Postprocedural coronary angiography demonstrated no critical coronary artery stenosis, compression, or flow limitation. Repeated venography showed no venous dissection.

The patient was discharged uneventfully. At 6-month follow-up, implantable cardioverter-defibrillator interrogation revealed no recurrent VT episodes. 24-hour Holter monitoring demonstrated approximately 500 multiform ventricular extrasystoles. Importantly, these ectopic beats exhibited a superior axis morphology, whereas the preablation clinical VA had demonstrated an inferior axis, suggesting successful modification of the dominant LVS substrate rather than persistence of the original focus.

## Discussion

Although RF ablation still remains the gold standard therapy for the VAs, it is far from perfection given that many scenarios such as LVS or intramural-epicardial substrates require bailout unconventional approaches such as bipolar ablation, needle ablation, half-saline ablation, and antegrade/retrograde ethanol administration into suitable branches. Ethanol ablation has emerged as a viable option recently. In a multicenter registry, acute success of retrograde ethanol was 98% and freedom from VAs at 1 year was 77%.^13^ However, there are still some significant caveats, such as inappropriate vascular anatomy to target leading to the inability to deliver ethanol to the desired area, insufficient myocardial damage owing to low dosage, or inadvertent damage to collateral tissue owing to downstream leakage through collaterals. In this case, we used a triple-balloon occlusion technique to prevent inadvertent damage to the anterior wall supplied by AIV. Double-balloon occlusion has been known to be used for large-bore venous posterolateral or middle cardiac vein branches^14^ but, to the best of our knowledge, this is the first case of triple-balloon retrograde ethanol administration for an LVS VA.

LVS arrhythmias are frequently challenging owing to deep intramural substrates.^15^ Intramural venous mapping poses a valuable window of opportunity for understanding the origin of LVS VAs. Unipolar wire recordings and slim multielectrode catheters can also be used to map coronary arteries or deep septal veins.^16^^,^^17^ Although this approach can enhance precision, owing to the lack of resources, targeting in this case was guided by detailed endocardial activation mapping and venographic-anatomic correlation.

Delivery of sufficient ethanol concentration over target site is another challenge. Although the exact number and volume of each ethanol application have not been standardized, most of the studies generally used 1 mL of ethanol and repeated the applications 3 times until myocardial staining with contrast was completed.^14^^,^^18^ Owing to the fact that our case was a recurrent one despite extensive ablation, we opted to use 3 × 2 mL of ethanol injections. Because ethanol is highly toxic to viable tissue, it is imperative that every caution should be taken to prevent inadvertent collateral damage. Sometimes, the target may be a large-bore vein supplying a broad area or one may find collaterals or interconnecting tributaries between the target and other major vein branches, precluding a single-balloon technique. These cases have been previously addressed with a double-balloon technique successfully.^12^^,^^14^ However, our case had both a large annular vein and a collateral, so we opted to use a triple balloon to restrict and intensify the alcohol effect to a predefined limited area.

At 6 months, the absence of recurrent VT and the morphologic shift from inferior to superior axis ventricular ectopy suggest durable modification of the dominant arrhythmogenic substrate. The residual multiform extrasystoles likely represent independent ectopic activity rather than persistence of the original LVS focus.

## Conclusion

Retrograde venous ethanol ablation has been demonstrated to be a very effective option in selected challenging bailout VA cases resistant to extensive RF. A detailed understanding of the venous anatomy is crucial for safe and effective implementation of this bailout technique.

## Disclosures

The authors have no conflicts of interest to disclose.
