# Supplementary material for: Triple-balloon occlusion for targeted retrograde coronary venous ethanol ablation of left ventricular summit ventricular tachycardia
Source: HeartRhythm Case Rep. 2026 Mar 24;12(6):680–4. doi: 10.1016/j.hrcr.2026.03.015 (PMC13270959; doi:10.1016/j.hrcr.2026.03.015)
Supplement: Supplementary File [file mmc2.docx]

An anonymized video demonstrating the venous anatomy has been uploaded as supplementary material.
